# Diagnostic Yield of EBUS Mediastinal Cryobiopsy Versus TBNA in Suspected Lymphoproliferative Disorders: A Prospective Multicentre Study

**DOI:** 10.1002/resp.70274

**Published:** 2026-06-17

**Authors:** Yaniv Dotan, Ayal Romem, Elad Guber, Michael Kassirer, Daphne Felus, Nimrod Urtreger, Ophir Freund, Hanna A. Dawood, Boaz Tiran, Doron Cohn‐Schwartz, Sarah Borsekofsky, Amir Bar‐Shai, Evgeni Gershman

**Affiliations:** ^1^ Pulmonary Institute, Rambam Health Care Campus and Ruth and Bruce Rappaport Faculty of Medicine Haifa Israel; ^2^ Pulmonary Department Meir Medical Center Kfar Saba Israel; ^3^ Faculty of Medicine Tel Aviv University Tel Aviv Israel; ^4^ Pulmonary Division Soroka Medical Center, Faculty of Medicine, Ben Gurion University Beer Sheva Israel; ^5^ The Institute of Pulmonary Medicine, Tel‐Aviv Sourasky Medical Center Tel Aviv Israel; ^6^ Institute of Pathology, Tel‐Aviv Sourasky Medical Center Tel Aviv Israel

**Keywords:** bronchoscopy and interventional techniques, histology/cytology, lymphoma, pathology

## Abstract

**Background and Objectives:**

The diagnosis of mediastinal lymphoproliferative disorders (LPDs) is often challenging and requires histopathological assessment from large tissue samples. As a result, new diagnostic techniques have been introduced alongside the commonly used endobronchial ultrasound‐guided trans‐bronchial needle aspiration (EBUS‐TBNA). Among them, EBUS‐guided transbronchial mediastinal cryobiopsy (EBUS‐TBMC) has shown promising potential, although high‐quality evidence remains limited. The aim of the study was to assess the diagnostic yield of EBUS‐TBMC compared with EBUS‐TBNA in patients with suspected mediastinal LPDs.

**Methods:**

This prospective multicentre study enrolled patients with suspected mediastinal LPDs undergoing bronchoscopy with EBUS for mediastinal lymph node sampling. All participants had either B symptoms or a history of LPD. All patients underwent EBUS‐TBNA followed by EBUS‐TBMC from the same mediastinal lymph node. Final diagnoses were confirmed after a 6‐month follow‐up.

**Results:**

Fifty‐eight patients underwent EBUS, of whom 50 completed both EBUS‐TBNA and EBUS‐TBMC. There were eight cases of failed EBUS‐TBMC who were not included in the analysis. The most frequent diagnoses were LPDs (54%) and sarcoidosis (30%). EBUS‐TBNA and EBUS‐TBMC had overall diagnostic yield of 52% and 80%, respectively. Among non‐diagnostic EBUS‐TBNA samples, 80% were diagnostic with EBUS‐TBMC (odds ratio 4.89, 95% CI 1.76–13.6). For confirmed LPDs, diagnostic yield was 41% for EBUS‐TBNA and 85% for EBUS‐TBMC. No major complications during or following the EBUS‐TBMC were observed.

**Conclusion:**

EBUS‐TBMC was superior to EBUS‐TBNA and achieved a high diagnostic yield in patients with suspected mediastinal LPDs.

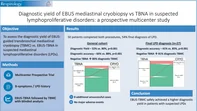

## Graphical abstract

We assessed the diagnostic yield of EBUS‐guided transbronchial mediastinal cryobiopsy (EBUS‐TBMC) compared with EBUS‐transbronchial needle aspiration (EBUS‐TBNA) in patients with suspected mediastinal lymphoproliferative disorders (LPDs). EBUS‐TBMC was superior to EBUS‐TBNA and achieved a high diagnostic yield in patients with suspected mediastinal LPDs. Our findings support integrating EBUS‐TBMC into clinical practice as a valuable tool in addition to TBNA for obtaining high‐quality tissue samples, particularly when LPDs are suspected, and histopathological architecture is essential for diagnosis. However, larger prospective studies are needed to further define the advantages, limitations, and optimal indications of EBUS‐TBMC.

## 1 Introduction

Endo‐bronchial ultrasound trans‐bronchial needle aspiration (EBUS‐TBNA) is the standard of care in the diagnosis and staging of lung cancer [1, 2], with reported sensitivities ranging from 69% to 95% and a low complication rate of 0.14% [3]. However, even though EBUS‐TBNA provides cytological material and, in some cases, cell‐block or clot‐core specimens suitable for limited histological assessment [4, 5], the samples are often insufficient for diagnosing mediastinal lymphoproliferative disorders (LPDs) [6] and other benign conditions which require histopathology analyses for a definitive diagnosis [7, 8].

Numerous strategies have been applied to increase the diagnostic yield of EBUS in LPDs, including the use of larger calibre core needles, adjunctive use of micro‐forceps biopsy, and, more recently, the use of cryoprobes for mediastinal lymph node sampling [9, 10, 11, 12]. Among these techniques, endobronchial ultrasound‐guided transbronchial mediastinal cryobiopsy (EBUS‐TBMC) has shown increased diagnostic yield [13, 14]. In EBUS‐TBMC, sampling is performed under direct EBUS visualization after a 1.1 mm cryoprobe is passed through the bronchoscope's working channel [15]. A systematic review and meta‐analysis comparing EBUS‐TBMC with EBUS‐TBNA in the diagnosis of lymphoma, which included 10 studies, found a statistically significant increase in the pooled diagnostic yield (87% vs. 29%, respectively, *p* = 0.001) with an odds ratio of 11.48 for a correct diagnosis [13].

While some evidence exists on the diagnostic yield of EBUS‐TBMC in cases suspicious for LPDs, as presented above, only five from the mentioned review specifically examined LPDs, and just two were prospective studies with very small patient samples [13, 15, 16]. Recently, Ariza‐Prota et al. [17] reported results from a multicentre retrospective study of 40 patients with lymphoma, further supporting the superior sensitivity of EBUS‐TBMC over EBUS‐TBNA (95% vs. 15%) in this population. Therefore, the objective of this study was to conduct a prospective multicentre interventional trial to evaluate the feasibility, diagnostic yield, and safety of EBUS‐TBNA versus EBUS‐TBMC for mediastinal lymph node biopsy in patients with suspected LPDs.

## 2 Methods

A prospective multicentre trial was conducted across pulmonary institutes in three major medical centres in northern, central, and southern Israel. The study included patients with mediastinal lymphadenopathy and suspicion of LPDs who were referred to EBUS‐TBNA in one of the included centres. To be eligible, patients had to meet the following criteria: (1) age 18 years or older, (2) ability to provide informed consent, (3) presence of mediastinal lymphadenopathy (> 1 cm in the shortest anterior–posterior axis) on thoracic computed tomography (CT), and (4) either ≥ 1 B‐symptom (fever, weight loss, or night sweats) or a known history of LPDs without an alternative explanation for lymph node enlargement (e.g., infection or other malignancy). Exclusion criteria were pregnancy, uncorrectable bleeding diatheses, and lack of respiratory or hemodynamic stability on the day of the procedure. Having a PET‐CT scan was not obligatory for inclusion.

This study was intentionally designed to include patients with a high pre‐test probability for LPD. High clinical suspicion of LPD was determined by the treating pulmonologist at each participating centre based on the above criteria. The study protocol did not mandate predefined PET‐CT uptake thresholds or standardized radiological scoring criteria. Imaging interpretation followed routine institutional practice. Enrollment did not require a mandatory haematology consultation prior to bronchoscopy, although cases were discussed in multidisciplinary settings according to local clinical practice.

The study was approved by the institutional review boards at all participating sites (0444‐22‐TLV, 0057‐23‐MMC, IRB‐0005‐23) and was registered in the Israeli Ministry of Health clinical trials database (MOH‐013103). The study was conducted in accordance with the Declaration of Helsinki and the protocol. Before every procedure at each centre, participants provided written informed consent to participate in the study and undergo both EBUS‐TBNA and EBUS‐TBMC, with the second performed for the purpose of this study. Patients who declined to consent were excluded.

### 2.1 Procedure

All patients underwent clinical evaluation by a senior pulmonologist prior to the procedure. EBUS procedures were performed under deep sedation, as previously described [18]. Topical anaesthesia with 2% lidocaine was administered—2 mL sprayed on the vocal cords and 2 mL on the main carina. Intravenous midazolam, fentanyl, and propofol were used for sedation, with dosing at the discretion of the sedating physician. After achieving deep sedation, a laryngeal mask airway (LMA) was inserted, and its position was verified with a diagnostic bronchoscope.

All procedures were performed by specialists in interventional bronchoscopy, each with over 5 years of experience in EBUS and hundreds of prior procedures. An EBUS bronchoscope (EVIS, BF‐UC190F, Olympus Medical Systems, Japan) was inserted through the LMA. Targeted lymph nodes were sampled first using a 21G TBNA needle (ViziShot 2, NA‐U401SX, Olympus Medical Systems, Japan). Each lymph node was sampled with at least three punctures, with 10–15 needle passes per puncture, according to the study protocol. Sampling began with the subcarinal lymph node (station 7) via the right or left main bronchus. If station 7 was inaccessible or unsuitable for sampling, the right para‐tracheal node (station 4R) was targeted. In cases where neither station 7 nor 4R could be sampled, the left or right hilar lymph nodes (stations 11L/11R, respectively) were selected, following the predefined protocol. There was no rapid on‐site cytological examination (ROSE) available at any site.

TBNA samples were preserved in 4% formaldehyde for histopathological analysis by an attending cytologist. At the cytologist's discretion, all samples underwent immunohistochemical staining, followed by flow cytometric immunophenotyping and additional cellular diagnostic and prognostic assessments, including FISH, IGH gene rearrangement, and SNP array analysis. These ancillary studies, including flow cytometry, were chosen based on cytological findings and clinical context and were not uniformly applied to all samples.

Following the final needle pass, the needle sheath was advanced while the needle remained in place within the lymph node, thereby dilating the trans‐bronchial tract. After this dilation step, the needle was withdrawn, and the EBUS scope was kept in contact with the airway wall to maintain visualization of the target lymph node. A 1.1 mm flexible cryoprobe (Erbecryo, 20402‐401, Erbe Elektomedizin, Germany) was then inserted through the working channel of the EBUS bronchoscope and guided into the tract created by the TBNA needle. The cryoprobe's position within the lymph node was confirmed sonographically. Cryo‐adhesion was activated for 6–8 s, after which the cryoprobe and bronchoscope were retracted together as a single unit (‘en bloc’) from the airway. All procedures were performed without the use of an ultrasound balloon at the scope tip. This extraction was performed entirely through the LMA, which acted as a protective sheath for the vocal cords, ensuring that the bulk tissue sample did not meet the laryngeal structures during removal. This step was repeated 2–3 times per sampled lymph node. Cryobiopsy samples were also preserved in 4% formaldehyde, with a similar analysis to TBNA, as mentioned above. All procedures were performed across participating centres according to the predefined study protocol, with inherent inter‐operator variability.

Given that EBUS‐TBMC is not yet an established routine technique and involves dilation of the bronchial wall with creation of a transbronchial tract, antibiotic prophylaxis was incorporated into the study protocol due to concerns regarding mediastinitis. Prophylaxis was standardized across all participating centres. Patients received oral ciprofloxacin 500 mg once daily for 3 days following the procedure. Ciprofloxacin was selected because of its broad‐spectrum coverage against common oropharyngeal and respiratory flora [19] and favourable oral bioavailability [20]. Post‐procedure, patients remained for 3 h per protocol for monitoring, including vital signs every half an hour. Patients were discharged after a senior pulmonologist reviewed their clinical status and chest x‐ray that was performed per protocol to exclude pneumothorax or pneumomediastinum. Patients were followed by a phone call up to 48 h after the procedure to evaluate complications. In addition, complications were assessed and recorded in the follow‐up visit, which occurred 2–4 weeks after the procedure.

### 2.2 Study Variables

Data collected include age, sex, symptoms, prior history of lymphoma, lymph node stations sampled, EBUS‐assessed lymph node size, and complications. Procedural complications included hypoxia, defined as an event of saturation < 90% for more than 30 s, hypotension that required pharmacological treatment such as intravenous fluids or vasopressors, airway bleeding with grading defined by CTACE criteria [21], or any other intervention to pause or stop the procedure at any stage. Post‐procedural complications were hypoxia of saturation longer than 30 s, hypotension that required any intervention, pneumothorax or pneumomediastinum as evident on post‐procedural chest x‐ray, fever of more than 38 C, or death.

Cytological and histological samples collected from EBUS‐TBNA and EBUS‐TBMC, respectively, were analysed by different pathologists who were blinded to the second pathological result and performed completely separately. Reports were obtained and compared for the target lymph node that was sampled by both modalities. Each pathological/cytological assessment was categorized by the research cytologists/pathologists as one of the following: (1) Diagnostic—defined as a sample with preserved tissue architecture and sufficient cellularity to allow for a definitive pathological diagnosis. This included the specific subtyping of haematological malignancies, identification of malignant epithelioid lineages, or definitive non‐malignant pathology (e.g., non‐caseating granulomas characteristic of sarcoidosis). (2) Non‐diagnostic: all other results, including samples with inadequate cellularity, extensive crush artefact, or non‐specific findings (e.g., blood only), that did not allow for a definitive diagnosis. Final diagnoses were adjudicated by a multidisciplinary discussion (MDD) at each participating centre, without knowledge of the sampling method used to obtain each result. Cases of discordant sampling results were resolved by the MDD based on clinical features, radiologic findings, and confidence in the histologic findings, with specific cases referred for additional sampling. For this trial, all patients were followed for at least 6 months post‐bronchoscopy to assess late complications, validate the EBUS diagnosis, and evaluate for subsequent diagnostic procedures performed. Only samples without a change in their final diagnosis at 6 months (compared with their pathological analysis) were ultimately defined as diagnostic.

### 2.3 Statistical Analysis

Diagnostic yield was the primary outcome and was defined as the proportion of cases in which the biopsy procured a pathological (malignant or benign) result that was diagnostic, without the need for additional biopsy. This outcome was assessed both for all completed (per protocol) and for all procedures, including uncompleted ones (intention‐to‐diagnose). Diagnostic accuracy of each EBUS sampling method was defined as the proportion of cases in which the sampling modality result was concordant with the final diagnosis established by multidisciplinary adjudication and 6‐month follow‐up [22]. A sub‐analysis of patients with a final diagnosis of LPD was also performed. Based on prior research [13], we conservatively assumed an overall diagnostic yield of 45% by EBUS‐TBNA and 75% by EBUS‐TBMC. Considering the above, to achieve a two‐sided alpha of 0.05 and a power of 80%, 42 patients were required. To account for variability in final diagnoses (which could affect the diagnostic yield) and for a 10% failure to complete the EBUS‐TBMC, a sample size of 55 patients was required. Procedures in which the TBNA and/or TBMC could not be performed due to technical or other reasons were counted but excluded from final analysis as the intent was to compare the actual samples retrieved.

Paired comparisons using related‐samples McNemar Change tests were used to compare the procedures' outcomes. Categorical variables were presented as sum (percentage of total). Continuous variables were presented as either mean ± standard deviation (SD) or median (interquartile range, IQR) based on their distribution (assessed by Kolmogorov Smirnov tests). Predictors for diagnostic EBUS‐TBNA were analysed using binary multivariate logistic regression, including significant associations from univariate analysis and sex and age as important baseline characteristics. All analyses were performed in SPSS version 29, with *p* < 0.05 for statistical significance.

## 3 Results

A total of 58 patients underwent EBUS with lymph node sampling. Eight cases (14%) were excluded due to unsuccessful cryoprobe insertion into the tunnel created by the EBUS‐TBNA. Therefore, 50 patients were included in the study cohort. The cohort's characteristics are shown in Table 1. The mean ± SD age was 61 ± 15 years, and 26 (52%) were female. Eighteen patients (36%) had a prior medical history of lymphoma. Reported B symptoms included fever (22%), weight loss (58%), and night sweats (36%). The subcarinal lymph node (station 7) was most commonly sampled (72%). The median (IQR) size of lymph nodes sampled, as measured by EBUS, was 22 mm (15–26 mm).

**TABLE 1:** Cohort characteristics.

Variable	Cohort *n* = 50 (%)
Age, years, mean ± SD	61 ± 15
Female sex	26 (52%)
Prior lymphoma	18 (36%)
B symptoms	
Fever	11 (22%)
Weight loss	29 (58%)
Night sweats	18 (36%)
Sampled lymph node	
Node 7	36 (72%)
Node 4R	14 (26%)
Node 11R	5 (10%)
Node 11L	5 (10%)
EBUS estimated lymph node size, mm, median (IQR)	22 (15–26)

The eight patients in whom cryoprobe insertion was unsuccessful had a mean age of 56.6 years, and 62.5% were women. Two patients (25%) had a prior history of lymphoproliferative disorder. The most frequently sampled lymph node station in this group was station 7 (50%), with a median lymph node size of 15 mm (10.7–18.5 mm). Technical failure was attributed to the inability to advance the cryoprobe through the TBNA‐created tract.

**FIGURE 1:**
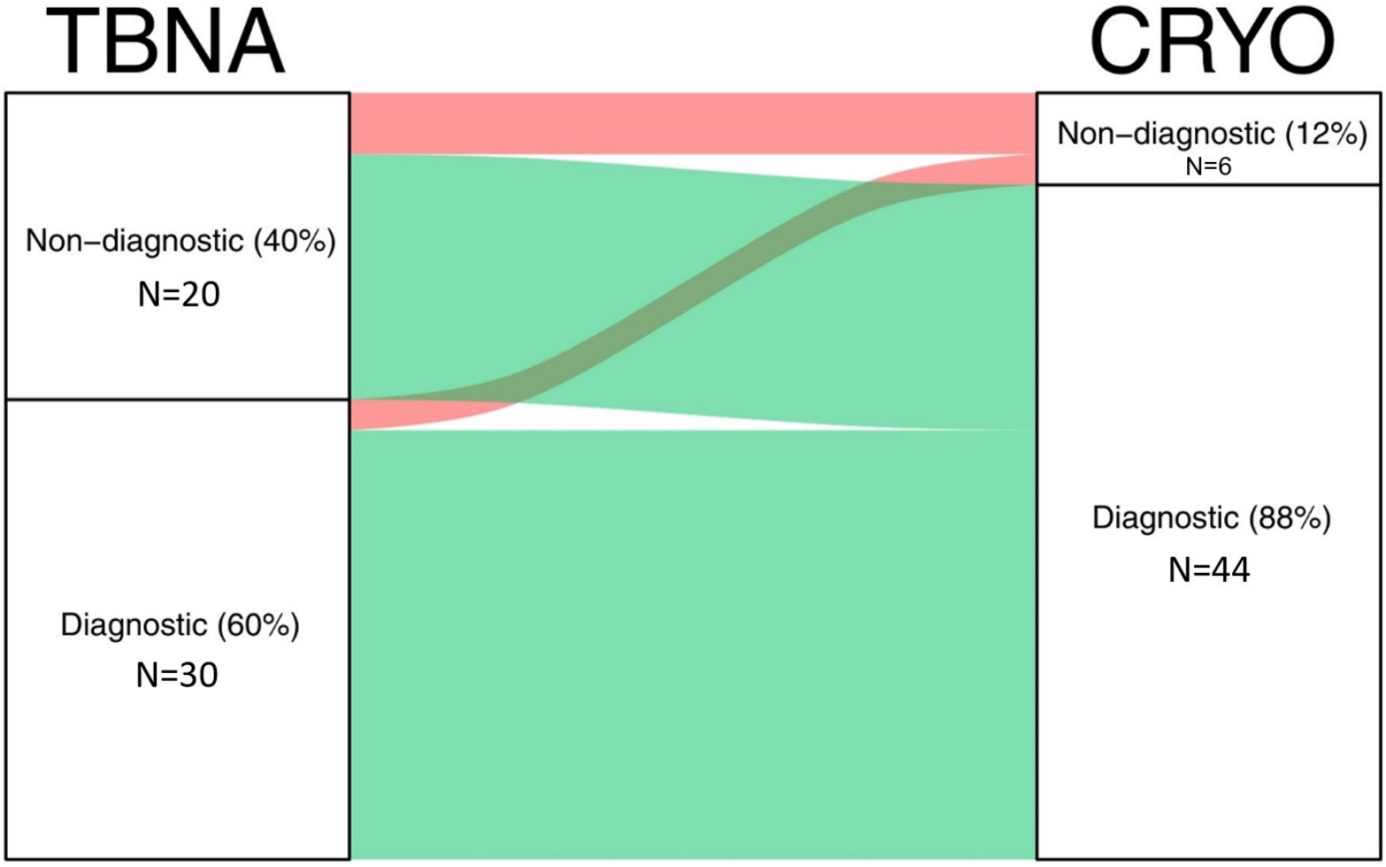
Results of trans‐bronchial needle aspiration (left) and trans‐bronchial cryobiopsy (right) in the study cohort.

Study outcomes are shown in Figure 1 and Table 2. The final diagnosis was LPDs in 54% of the cases, with follicular lymphoma as the most common diagnosis (9 cases). EBUS‐TBNA had a diagnostic accuracy of 60%, compared to 88% using EBUS‐TBMC (*p* < 0.01). The diagnostic yield of EBUS‐TBNA was 52%, while it was 80% using EBUS‐TBMC (*p* < 0.01). Including the eight failed TBMC cases, EBUS‐TBMC had a diagnostic yield of 69% and a diagnostic accuracy of 76%. Of the 20 cases without diagnostic EBUS‐TBNA results, 16 (80%) had a diagnostic EBUS‐TBMC (OR 4.89, 95% CI 1.76–13.6, *p* < 0.01), contributing to an additional 32% of diagnostic results by the bronchoscopy. Two cases of diagnostic EBUS‐TBNA had non‐diagnostic TBMC.

**TABLE 2:** Study outcomes.

Variable	Total, *n* = 50 (%)
Final diagnosis	
Lymphoproliferative disorder	27 (54%)
Follicular lymphoma	9 (18%)
Hodgkin's lymphoma	6 (12%)
Marginal zone lymphoma	4 (8%)
Diffuse large B‐cell lymphoma	3 (6%)
T‐cell lymphoma	2 (4%)
Mantle cell lymphoma	1 (2%)
Small lymphocytic lymphoma	1 (2%)
Castleman's disease	1 (2%)
Non‐hematologic	23 (46%)
Sarcoid	15 (30%)
Carcinoma	4 (8%)
Reactive lymph node	4 (8%)
Complications	
Bleeding (Grade 1)	5 (10%)
Bleeding (Grade 2)	3 (6%)
Bleeding (Grade 3–4)	0 (0%)
Pneumothorax	0 (0%)
Fever	4 (8%)
Hypotension	2 (4%)
Transient hypoxemia	6 (12%)
Procedure‐related death	0 (0%)

Among the four cases with non‐diagnostic results from both EBUS‐TBNA and EBUS‐TBMC, mediastinoscopy was performed in three patients. The patients included two females (aged 60 and 48 years) and one male (aged 77 years). All three were ultimately diagnosed with a lymphoproliferative disorder (Table 3). The fourth case was lost to follow‐up, and a final diagnosis was unknown. Importantly, among patients with diagnostic EBUS‐TBNA and/or EBUS‐TBMC, no additional invasive diagnostic procedures were required for tissue acquisition, including for lymphoma subtyping, immunophenotyping, or molecular analyses.

**TABLE 3:** Characteristics of patients requiring mediastinoscopy for final diagnosis.

Patient	Age	Sex	EBUS‐TBNA result	EBUS‐cryo result	Final diagnosis
1	60	Female	High grade B cell lymphoma	Suspicious for B cell lymphoma	Marginal zone lymphoma
2	48	Female	Normal lymph node	Normal lymph node	Castleman's disease
3	77	Male	Atypical lymphocytes	Normal lymph node	Diffuse large B cell lymphoma

Procedural complications are shown in Table 2. Grade 1 and 2 bleeding occurred in eight cases (16%), all after TBNA. No additional bleeding followed TBMC. Four patients (8%) had transient fever in the days following the procedure without any infectious sequelae. Importantly, although fever was included among the B‐symptoms used for eligibility, no patients had active fever at the time of the procedure. Therefore, all post‐procedural fever events represented new‐onset fever following bronchoscopy. During the procedure, two patients (4%) suffered from hypotension requiring pharmacological treatment, and six patients (12%) had transient hypoxemia. All recovered well without the need for any additional intervention. No deaths occurred within 30 days post‐procedure, except one unrelated suicide.

### 3.1 Performance in LPD and Non‐LPD Subgroups

**FIGURE 2:**
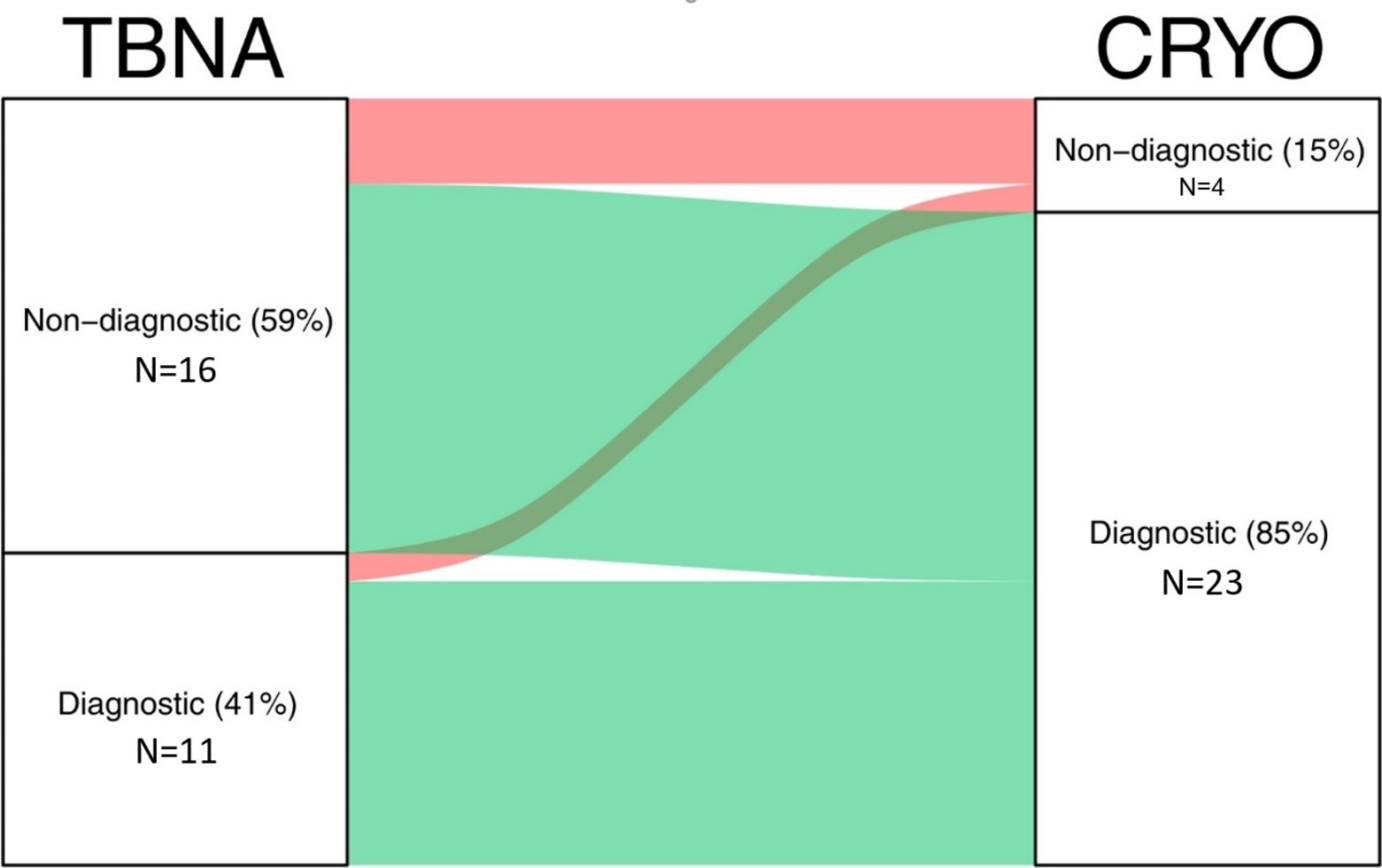
Results of trans‐bronchial needle aspiration (left) and trans‐bronchial cryobiopsy (right) in patients with a final diagnosis of lymphoproliferative disease.

We performed a subgroup analysis comparing patients with a final diagnosis of LPD (*n* = 27, 54%) and those with non‐LPD diagnoses (*n* = 23, 46%), the latter comprising sarcoidosis (*n* = 15), carcinoma (*n* = 4), and reactive lymphadenopathy (*n* = 4). In the LPD subgroup, EBUS‐TBNA provided diagnostic results in 41% of the cases, compared to 85% by the EBUS‐TBMC (Figure 2). Notably, among the 16 LPD cases without a definite diagnosis by EBUS‐TBNA, 13 (81%) were diagnostic by TBMC, contributing to an additional 48% of diagnostic yield by bronchoscopy. In addition, there was no difference in the diagnostic yield of EBUS‐TBNA between those with a prior diagnosis of LPD (39%) and those without (44%; *p* = 0.782).

In the non‐LPD subgroup, EBUS‐TBMC diagnostic yield was 74%, compared to 65% with EBUS‐TBNA. Diagnostic accuracy in that group was 91% for EBUS‐TBMC and 82% for EBUS‐TBNA. Sarcoidosis was the most common diagnosis in this group (*n* = 15, 65%) and had a high diagnostic yield for both modalities, with EBUS‐TBMC at 87% and EBUS TBNA at 73%. These results highlight that while TBNA performs relatively well in non‐LPD conditions, its yield is substantially lower in LPD cases (41%). Of note, there was no statistically significant difference in lymph node size between patients with a final diagnosis of sarcoidosis and those with LPDs (median [IQR] 20 mm [15–25] vs. 24 mm [16–40], *p* = 0.178).

Analysis of predictors for diagnostic EBUS‐TBNA is shown in Table 4. Sampling from the sub‐carinal lymph node was significantly associated with a higher likelihood of obtaining a diagnostic result (OR 4.09, 95% CI 1.11–15.1, *p* = 0.034). In contrast, a final diagnosis of LPD was associated with a non‐diagnostic TBNA outcome (OR 0.145, 95% CI 0.04–0.54, *p* < 0.01).

**TABLE 4:** predictors for diagnostic trans‐bronchial needle aspiration.

Variable	OR	95% CI	*p*
Age	0.993	0.96–1.03	0.72
Female sex	0.626	0.20–1.96	0.42
Sub‐carinal lymph node	4.091	1.11–15.1	0.03
Lymph node size	0.996	0.96–1.03	0.83
Lymphoproliferative diagnosis	0.145	0.04–0.54	< 0.01

## 4 Discussion

This prospective multicentre study assessed EBUS‐TBMC diagnostic yield and safety in suspected mediastinal LPDs. In our study, EBUS‐TBMC significantly outperformed EBUS‐TBNA in both its diagnostic yield and accuracy. The differences were even more pronounced in cases with a final diagnosis of LPD. There were no severe adverse events or complications during or after completion of our study. Notably, EBUS‐TBMC was not performed in 14% of cases due to technical limitations, which likely introduced bias to our results.

Patients with suspected mediastinal LPDs present a diagnostic challenge. Haematologists and pathologists require a large sample of the involved tissue, usually a lymph node, that can provide tissue architecture for a definite diagnosis. Historically, mediastinoscopy was used to obtain such large samples [23]. Mediastinoscopy is generally safe, does not require routine hospitalization or antibiotic prophylaxis, and is associated with low complication rates when performed in experienced centres [24, 25]. Still, it is a surgical procedure requiring general anaesthesia and operating room resources, and in some cases may cause complications such as bleeding, pneumothorax, and wound infection [1, 24, 26]. Therefore, other modalities were utilized over the past years, trying to replace mediastinoscopy with less invasive procedures [9, 10, 11].

Among the different options for non‐surgical mediastinal sampling, TBMC has gained popularity due to its ability to obtain relatively large, well‐preserved tissue samples suitable for histopathologic [16] and immunophenotypic analysis [27, 28, 29]. These technical advantages are especially important in conditions requiring preserved tissue architecture. Lymphoproliferative disorders require adequate nodal architecture for histopathological classification, immunophenotyping, and molecular studies, which are often not achievable with cytology‐based techniques such as TBNA [30, 31]. In contrast, granulomatous diseases such as sarcoidosis are characterized by well‐formed, non‐caseating granulomas that are often identifiable on cytological specimens, contributing to the relatively high diagnostic yield of TBNA in this setting [32]. Similarly, metastatic carcinoma is frequently identifiable on cytology due to the presence of malignant epithelial cells. These biological differences likely explain why the incremental benefit of TBMC is most pronounced in LPDs, whereas it is more modest in non‐LPD conditions.

A large meta‐analysis and systematic review comparing EBUS‐TBMC with TBNA for the diagnosis of lymphoma demonstrated a significantly higher pooled diagnostic yield with TBMC (87% vs. 29%, *p* = 0.001; OR of 11.48) [13], consistent with our results. Notably, unlike most studies included in the meta‐analysis, our results are derived from a prospective multicentre study conducted in real‐world clinical settings that enrolled patients specifically suspected of having LPDs. Additional prior cohorts reported similar diagnostic yields for EBUS‐TBNA, ranging from 15% to 50% [15, 16, 17]. Even when including the eight cases in which EBUS‐TBMC was not feasible, TBMC still outperformed EBUS‐TBNA and successfully avoided the need for mediastinoscopy in the majority of cases.

The diagnostic yield of EBUS‐TBNA in our cohort (48%) was lower than that reported in some previous series, which could impact the observed overall superiority of EBUS‐TBMC found in our study. This likely reflects the enrichment of our population with suspected LPDs, a setting in which TBNA is known to perform less well due to the requirement for preserved nodal architecture and larger tissue samples [30, 31]. Consistently, subgroup analysis demonstrated lower TBNA yield in LPD cases compared with non‐LPD conditions such as sarcoidosis. Procedural factors may also have contributed: although multiple passes were performed, rapid on‐site evaluation was unavailable, ancillary studies (e.g., flow cytometry) were not standardized, and inter‐centre variability in technique and cytopathologic processing may have further influenced TBNA performance. In addition, 36% of patients had a prior history of LPDs. Prior studies suggest TBNA may have a higher yield in relapsed disease [17, 33]. However, we did not find such a difference, possibly due to the small sample size in this sub‐analysis.

Another interesting observation was the high sarcoidosis rate among suspected LPDs. Sarcoidosis is a systemic disease that involves the lungs in about 90% of cases [32]. Its clinical and radiological presentations are highly variable, ranging from asymptomatic mediastinal lymphadenopathy to a multisystemic disease with constitutional symptoms such as weight loss, fever, and night sweats—collectively known as B‐symptoms [34]. These manifestations are also common in LPDs [35], making the differential diagnosis particularly challenging and necessitating tissue sampling for definitive diagnosis. In our study, of the 15 patients diagnosed with sarcoidosis, 73% had diagnostic samples obtained via EBUS‐TBNA, while EBUS‐TBMC yielded diagnostic tissue in 87% of cases.

In our cohort, TBMC could not be performed in 8 out of 58 attempted procedures due to technical difficulties. Nevertheless, the overall complication rate was low, without cases of pneumothorax, pneumomediastinum, mediastinitis, or procedural‐related death. It should be noted that all patients received standardized post‐procedural antibiotic prophylaxis as part of the study protocol. Therefore, the absence of infectious complications may partially reflect this practice, and safety outcomes should be interpreted within this context. These findings may not be directly generalizable to settings where prophylactic antibiotics are not routinely administered. Routine fluoroquinolone prophylaxis, as done in our trial, may not be appropriate in regions where tuberculosis is endemic due to association with development of drug‐resistant tuberculosis. Compliance with local guidelines should be practiced. In addition, a post‐procedural chest X‐ray was systematically performed in all patients to evaluate for complications such as pneumothorax or pneumomediastinum. While this approach was adopted to ensure standardized safety monitoring, routine imaging is not universally recommended following conventional EBUS procedures and may not be required in all real‐world settings. EBUS‐TBMC has previously been shown to be safe, with minor bleeding as the main complication [13, 36]. This strengthens the potential role of EBUS as a first step in evaluating mediastinal lymphadenopathy suspicious for LPDs.

Our study has several limitations. First, although this is one of the largest prospective studies of patients with suspected LPDs, the sample size remains relatively small, limiting the power for subgroup analyses. Second, specimen analysis was performed by pathologists at each participating centre rather than through a centralized review, which might have introduced inter‐observer variability in interpretation. Third, the final diagnosis was made by the tumour board at each centre, which may have contributed to a lack of standardization, although all cases were subsequently reviewed by the research team to enhance reliability. Fourth, EBUS‐TBMC was performed after TBNA and through the needle tract. This fixed procedural sequence may have introduced a sequence bias. This design was required to facilitate cryoprobe insertion; however, prior needle sampling may have altered tissue characteristics or influenced subsequent cryobiopsy yield. Fifth, the use of a cohort with a high pre‐test probability for LPDs may limit extrapolation of these findings to populations with lower disease prevalence. This might reflect the lower overall diagnostic yield of EBUS‐TBNA compared with some prior studies and should be considered when interpreting the comparison between the two diagnostic methods. Additionally, PET CT characteristics were not systematically available, limiting the assessment of pre‐test probability and procedural targeting, potentially influencing the observed diagnostic yield. Furthermore, we did not systematically assess quantitative tissue quality parameters (e.g., specimen volume, crush artefact, or nucleic acid yield), and the adequacy of ancillary studies (e.g., flow cytometry and immunohistochemistry) in a standardized manner across centres. Finally, the results may not be generalizable to centres with limited access to cryobiopsy tools or technical expertise.

In conclusion, our prospective multicentre study demonstrates that EBUS‐TBMC offers a higher diagnostic yield and accuracy in patients with mediastinal lymphadenopathy suspected of LPDs, compared to EBUS‐TBNA. The procedure demonstrated a favourable safety profile with low complication rates, although the 14% failure rate to perform TBMC should be considered. These findings support integrating EBUS‐TBMC into clinical practice as a valuable tool in addition to TBNA for obtaining high‐quality tissue samples, particularly when LPDs are suspected, and histopathological architecture is essential for diagnosis. However, larger prospective studies are needed to further define the advantages, limitations, and optimal indications of EBUS‐TBMC.

## Author Contributions

**Yaniv Dotan:** conceptualization, data curation, supervision, writing – original draft. **Ayal Romem:** conceptualization, methodology, data curation, supervision, writing – original draft. **Elad Guber:** conceptualization, data curation, investigation, writing – review and editing. **Michael Kassirer:** conceptualization, validation, methodology, writing – review and editing. **Daphne Felus:** methodology, validation, formal analysis, writing – original draft. **Nimrod Urtreger:** data curation, formal analysis, writing – original draft. **Ophir Freund:** formal analysis, visualization, writing – original draft. **Hanna A. Dawood:** writing – review and editing, data curation. **Boaz Tiran:** data curation, writing – review and editing, investigation. **Doron Cohn‐Schwartz:** data curation, writing – review and editing. **Sarah Borsekofsky:** investigation, validation, writing – review and editing. **Amir Bar‐Shai:** supervision, investigation, writing – review and editing, resources. **Evgeni Gershman:** conceptualization, methodology, data curation, writing – original draft, project administration. [Correction added on 30 June 2026, after first online publication: The author’s name “Doron Cohn Shwartz” has been corrected to “Doron Cohn‐Schwartz.”]

## Funding

This study was supported in part by grant no. 3000‐18747 from the Chief Scientist Office of the Ministry of Health, Israel. In addition, the study was partially funded by the Israel Lung Association, Tel‐Aviv.

## Ethics Statement

The study was conducted in accordance with the declaration of Helsinki, with approval by the Tel Aviv Sourasky Medical Center review board (0444‐22‐TLV), the Rambam Health Care Campus review board (0005‐23), and Meir Medical Center review board (0057‐23‐MMC).

## Conflicts of Interest

The authors declare no conflicts of interest.

## Data Availability

The data that support the findings of this study are available from the corresponding author upon reasonable request.
